# Alternate day fasting combined with a low‐carbohydrate diet for weight loss, weight maintenance, and metabolic disease risk reduction

**DOI:** 10.1002/osp4.367

**Published:** 2019-09-13

**Authors:** Faiza Kalam, Kelsey Gabel, Sofia Cienfuegos, Eric Wiseman, Mark Ezpeleta, Malik Steward, Vasiliki Pavlou, Krista A. Varady

**Affiliations:** ^1^ Department of Kinesiology and Nutrition University of Illinois at Chicago Chicago Illinois

**Keywords:** alternate day fasting, body weight, low‐carbohydrate diet, obesity

## Abstract

**Objective:**

Alternate day fasting (ADF) is a popular weight loss regimen. Whether carbohydrate restriction can enhance the weight loss achieved with ADF remains unclear. Accordingly, this study examined the effect of ADF combined with a low‐carbohydrate diet on body weight and metabolic disease risk factors.

**Methods:**

Adults with obesity (n = 31) participated in ADF (600 kcal “fast day” alternated with an ad libitum “feast day”) with a low‐carbohydrate background diet (30% carbohydrates, 35% protein, and 35% fat). The 6‐month trial consisted of a 3‐month weight loss period followed by a 3‐month weight maintenance period.

**Results:**

Body weight decreased (−5.5 ± 0.5%; *P* < .001) during the weight loss period (month 0‐3) but remained stable (*P* = .57) during the weight maintenance period (month 4‐6). Net weight loss by month 6 was −6.3 ± 1.0%. Fat mass was reduced (*P* < .01) by month 6, while lean mass and visceral fat mass remained unchanged. Total cholesterol and low‐density lipoprotein (LDL) cholesterol levels decreased (*P* < .05) by −6 ± 2% and − 8 ± 3%, respectively, by month 6. Systolic blood pressure was also reduced (*P* = .03) by −7 ± 3 mm Hg. Fasting insulin decreased (*P* = .03) by −24 ± 8% by month 6 relative to baseline. High‐density lipoprotein (HDL) cholesterol, triglycerides, diastolic blood pressure, heart rate, fasting glucose, homeostatic model assessment of insulin resistance (HOMA‐IR), and haemoglobin A1C (HbA1c) remained unchanged.

**Conclusions:**

These findings suggest that ADF combined with a low‐carbohydrate diet is effective for weight loss, weight maintenance, and improving certain metabolic disease risk factors such as LDL cholesterol, blood pressure, and fasting insulin. While these preliminary findings are promising, they still require confirmation by a randomized control trial.

## 1 INTRODUCTION

Obesity is associated with a host of comorbidities including coronary heart disease, dyslipidaemia, hypertension, and type 2 diabetes.^1^, ^2^ Accumulating evidence suggests that alternate day fasting (ADF) is an effective diet strategy to help individuals with obesity lose weight and lower metabolic disease risk. 3, 4, 5, 6 ADF regimens include a “feast day” where food is consumed ad‐libitum over 24 hours, alternated with a “fast day” where intake is limited to approximately 600 kcal over 24 hours. Recent ADF studies demonstrate that participants lose 3% to 7% of body weight after 2 to 12 months and experience improvements in lipid profiles, blood pressure, and insulin sensitivity.^7^, ^8^, ^9^, ^10^, ^11^, ^12^

While these preliminary findings are promising, each of these previous trials implemented a traditional high‐carbohydrate diet (50%‐60% energy as carbohydrates on the fast and feast days).^7^, ^8^, ^9^, ^10^, ^11^, ^12^ Mounting evidence indicates that individuals with obesity lose more weight when a low‐carbohydrate diet (approximately 30% energy as carbohydrate) is implemented.^13^, ^14^, ^15^ Low‐carbohydrate diets may facilitate weight loss by increasing subjective fullness, while decreasing hunger.^16^, ^17^ Some findings also suggest that low‐carbohydrate diets may improve metabolic disease risk indicators (low‐density lipoprotein [LDL] cholesterol, blood pressure, and fasting insulin) to a greater degree than high‐carbohydrate diets.^18^, ^19^, ^20^ However, the effects of a low‐carbohydrate diet during ADF have never been tested.

Evidence suggests that the incorporation of portion‐controlled liquid meals can enhance weight loss as these items help individuals stay within the confines of their prescribed energy goals.^21^ To this end, findings from a recent meta‐analysis suggest that meal replacements produce twice as much weight loss as traditional food‐only diet regimens.^22^ Whether the inclusion of portion controlled meals can help bolster weight loss during ADF remains unclear.

Accordingly, this study examined the effects of ADF combined with low‐carbohydrate meal replacements on body weight and metabolic disease risk factors in adults with obesity. This study hypothesized that subjects would lose 6%^7^, ^8^, ^9^, ^10^, ^11^, ^12^ of baseline body weight during the 3‐month weight loss period and maintain this weight loss during the 3‐month weight maintenance period. This study also hypothesized that blood pressure and fasting insulin would be significantly lowered by month 6 when compared with baseline.

## 2 METHODS

### 2.1 Subject selection

Subjects (n = 94) were recruited from the Chicago area by means of flyers placed on and around the University of Illinois, Chicago campus. Inclusion criteria were as follows: body mass index (BMI) between 30.0 and 49.9 kg/m^2^; age between 18 and 65 years; pre‐menopausal or postmenopausal (absence of menses for more than 2 years); lightly active (less than 3 h/wk of light intensity exercise at 2.5 to 4.0 metabolic equivalents [METs] for 3 months prior to the study); weight stable for 3 months prior to the beginning of the study (less than 4 kg weight loss or weight gain); nondiabetic; no history of cardiovascular disease (myocardial infarction or stroke); nonsmoker; not a night shift worker; and not taking weight loss medications. The experimental protocol was approved by the Office for the Protection of Research Subjects at the University of Illinois, Chicago. All volunteers gave written informed consent to participate in the trial. All methods were performed in accordance with the relevant guidelines and regulations stipulated by the Office for the Protection of Research Subjects at the University of Illinois, Chicago.

### 2.2 Experimental design

A 6‐month longitudinal study with a 1‐month baseline control period was implemented to test the study objectives. During the baseline control period, subjects were asked to maintain their weight by not changing their eating or activity habits. As such, each subject served as her or his own control. During the 6‐month ADF intervention, subjects participated in a 3‐month weight loss period followed by a 3‐month weight maintenance period.

### 2.3 ADF low‐carbohydrate diet

The 6‐month ADF low‐carbohydrate intervention was designed to have the following macronutrient composition: 30% energy from carbohydrates, 35% energy from protein, and 35% energy from fat. Meal replacements (Optifast HP Shake Mix, Nestle Health Sciences, Vevey, Switzerland) were provided on the fast and feast days to help subjects adhere to their calorie goals and attain macronutrient targets. The meal replacements were provided to subjects free of charge. Each meal replacement shake packet contained 26 g of protein, 10 g of carbohydrates, 6 g of fat, and 200 kcal. The fast and feast days began at midnight each day. An overview of the prescribed diet is portrayed in Table 1.

**Table 1:** Overview of the prescribed meal replacement regimen

	Weight Loss Period (Months 0‐3)		Weight Maintenance Period (Month 4‐6)	
Nutrients	Feast day^a^	Fast day^b^	Feast day^a^	Fast day^b^
Meal replacement packets	5	3	3	3
Energy, kcal	1000	600	600	600
Protein (% kcal, g)	53%, 130 g	53%, 78 g	53%, 78 g	53%, 78 g
Carbohydrates (% kcal, g)	20%, 50 g	20%, 30 g	20%, 30 g	20%, 30 g
Fat (% kcal, g)	27%, 30 g	27%, 18 g	27%, 18 g	27%, 18 g

a On feast days, subjects could consume food ad libitum after they had finished consuming all of their meal replacements. Counselling was provided to teach subjects how to select low carbohydrate foods to meet macronutrient targets (approximately 30% carbohydrates, approximately 35% protein, and approximately 35% fat).

b No other foods or beverages were permitted on the fast day (except of water, black coffee, and tea).

#### 2.3.1 Weight loss period (month 0‐3)

On the fast day, subjects consumed three meal replacements that provided a total of 600 kcal. No other foods or beverages were permitted on the fast day, with the exception of water, black coffee, and tea. On alternating feast days, subjects consumed five meal replacements that provided a total of 1000 kcal.

#### 2.3.2 Weight maintenance period (month 4‐6)

On the fast day, subjects continued to consume three meal replacements providing 600 kcal. No other foods or beverages were permitted on the fast day (except of water, black coffee, and tea). On alternating feast days during the maintenance period, subjects consumed three meal replacements that provided a total of 600 kcal.

### 2.4 Dietary counselling and ad libitum food intake on feast days

Subjects were permitted to eat food ad libitum on the feast day during the weight loss and weight maintenance periods after they had finished consuming all of their meal replacements. The study dietician provided one‐on‐one counselling every 2 weeks to teach subjects how to select low‐carbohydrate foods to meet macronutrient targets (approximately 30% carbohydrates, approximately 35% protein, and approximately 35% fat).

### 2.5 Meal replacement protocol adherence

Compliance with the meal replacement protocol was monitored using a daily “Shake adherence log.” The logs were collected and reviewed by study personnel at the weigh‐in every 2 weeks. Percent adherence was calculated separately for the fast day and feast day as follows:

$$\%\text{Adherence}=\left[\#\text{shakes consumed}/\#\text{of shakes distributed}\right]\times 100.$$

### 2.6 Body weight and body composition assessment

Body weight measurements were taken to the nearest 0.25 kg at baseline and every 2 weeks during the intervention in light clothing and without shoes using a digital scale (Omron HBF‐500; Omron Health Care, Lake Forest, IL). Height was assessed using a wall‐mounted stadiometer to the nearest 0.1 cm at baseline. Body composition (fat mass, lean mass, and visceral fat mass) was measured using dual X‐ray absorptiometry (DXA; iDXA, General Electric Inc) during the baseline period and at the end of months 3 and 6.

### 2.7 Dietary intake and physical activity assessment

All subjects were asked to complete a 7‐day food record during the baseline control period at the end of the weight loss period (month 3) and at the end of the weight maintenance period (month 6). The study dietitian provided 15 minutes of instruction to each participant on how to complete the food records. These instructions included information and reference guides on how to estimate portion sizes and record food items in sufficient detail to obtain accurate estimates of dietary intake. Subjects were not required to weigh foods but were asked to measure the volume of foods consumed with household measures (ie, measuring cups and measuring spoons). The food analysis program, Nutritionist Pro (Axxya Systems, Stafford, TX) was used to calculate the total daily intake of energy, fat, protein, carbohydrate, cholesterol, and fibre. All subjects were asked to maintain their level of physical activity throughout the trial. Activity level (steps/day) was measured over 7 days during the baseline control period and at the end of months 3 and 6 by a pedometer (Yamax Digi‐walker SW‐200, Yamax Inc, San Antonio, TX).

### 2.8 Metabolic disease risk factor assessment

All metabolic disease risk variables were measured at month 0 and at the end of month 3 and month 6. Twelve‐hour fasting blood samples were collected between 6:00 am and 9:00 am on the morning of a feast day. The subjects were instructed to avoid exercise, alcohol, and coffee for 24 hours before each visit. Fasting plasma total cholesterol, direct LDL cholesterol, HDL cholesterol, triglycerides concentrations were measured by a commercial lab (Medstar, Chicago, IL). Fasting glucose concentrations were measured with a hexokinase reagent kit (Abbott, South Pasadena, CA). Fasting insulin was assessed as total immunoreactive insulin (Coat‐A‐Count Insulin, Los Angeles, CA). Insulin resistance (IR) was calculated using the homeostatic model assessment (HOMA) method, by applying the following formula: [HOMA‐IR *=* fasting insulin (μlU/mL) × fasting glucose (mg/dL) /405]. Haemoglobin A1C (HbA1c) was assessed by enzyme‐linked immunosorbent assay (ELISA) (Mybiosource, San Diego, CA). Blood pressure and heart rate were measured in triplicate using a digital automatic blood pressure/heart rate monitor (Omron HEM 705 LP, Kyoto, Japan) with the subject in a seated position after a 10‐minute rest.

### 2.9 Statistics

For the sample size calculation, it was estimated that the ADF low‐carbohydrate intervention would reduce body weight by 6% by month 6. It was calculated that n = 29 participants would provide 95% power to detect a significant difference of 6% between baseline and month 6, using a two‐tailed paired samples *t* test with *α* = .05. A dropout rate of 25% was anticipated. Thus, the study initially aimed to recruit 39 participants assuming that 29 participants would complete the trial. It was later decided to recruit a total of 52 participants to increase statistical power, since the dropout rate was higher than expected. Results are presented as mean ± SEM. Normality was assessed by the Kolmogorov‐Smirnov test. No variables were found to be not normally distributed. Baseline characteristics were compared between completers and dropouts using independent samples *t* test (continuous variables) and the McNemar test (categorical variables). A repeated measures ANOVA was used to assess differences in outcome measures over time (baseline, month 3, and month 6). Post‐hoc analyses were performed using the Bonferroni correction. Differences were considered significant at *P* < .05. All data were analysed using SPSS software (version 25.0, SPSS Inc, Chicago, IL).

## 3 RESULTS

### 3.1 Baseline characteristics

**Figure 1:**
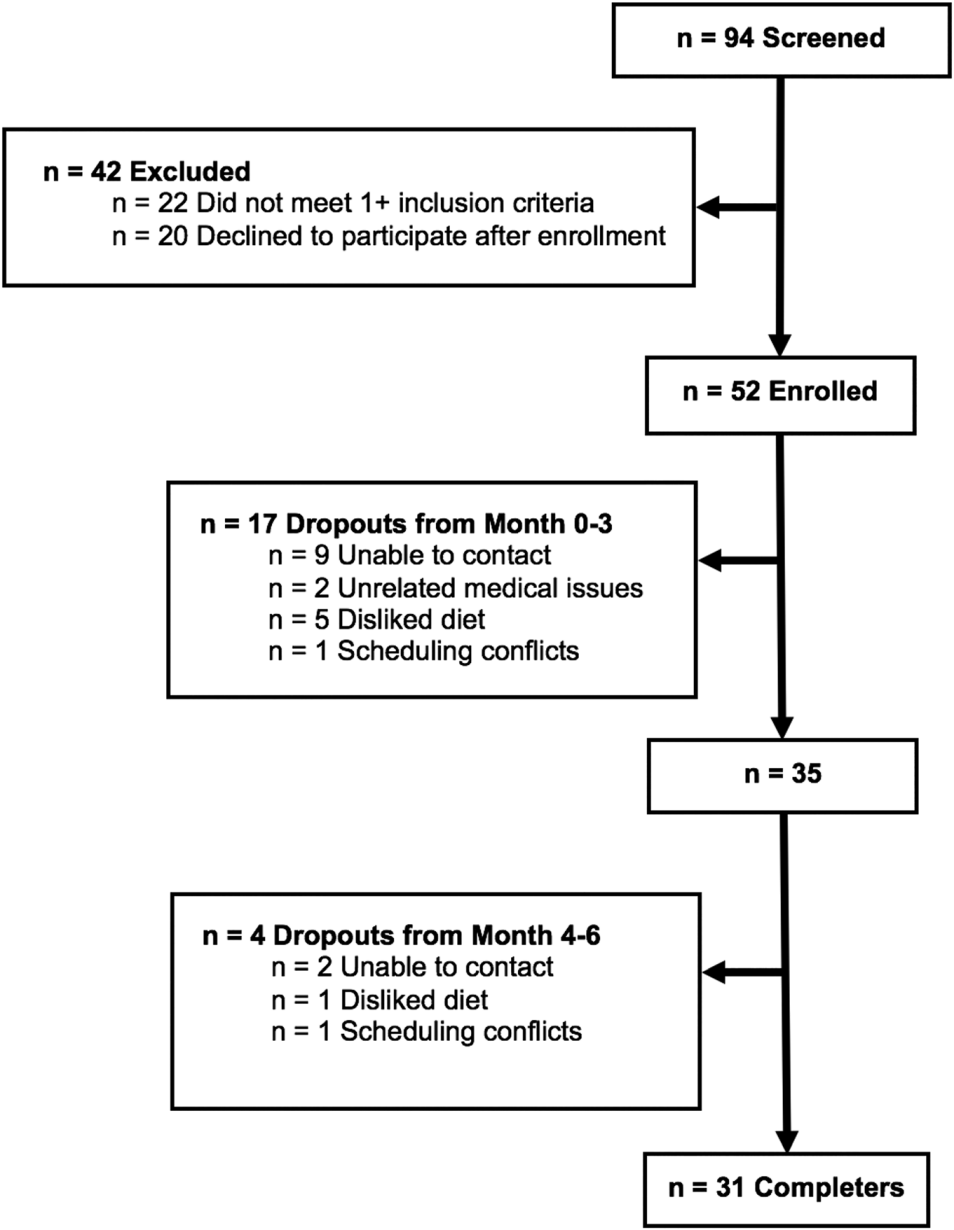
Study flow chart

A total of n = 94 individuals expressed interest in the study, n = 42 were excluded or declined to participate, and n = 52 began the ADF intervention (Figure 1). Seventeen subjects dropped out by the end of weight loss period (month 3), and four more dropped out by the end of the weight maintenance period (month 6). Thirty‐one subjects completed the full 6‐month protocol. Reasons for subject dropout included medical issues unrelated to the study (n = 2), scheduling conflicts (n = 2), dislike of the ADF protocol or the meal replacements (n = 6), or inability to contact (n = 11). There were no differences in baseline characteristics between completers and dropouts (Table 2).

**Table 2:** Baseline characteristics

Characteristic	Completer	Dropout	*P* value
n	31	21	
Age, y	48 ± 2	42 ± 2	.13
Sex (F/M)	25/6	19/2	.84
Race or ethnicity			
White	1	7	.30
Black	18	9	
Asian	3	1	
Hispanic	9	4	
Other	0	0	
Height, cm	163 ± 2	163 ± 2	.76
Weight, kg	100 ± 3	99 ± 4	.91
Body mass index, kg/m^2^	38 ± 1	37 ± 1	.72

*Note*. Values reported as mean ± SEM. *P* value: Independent samples *t* test (continuous variables); McNemar test (categorical variables).

### 3.2 Body weight and body composition

**Figure 2:**
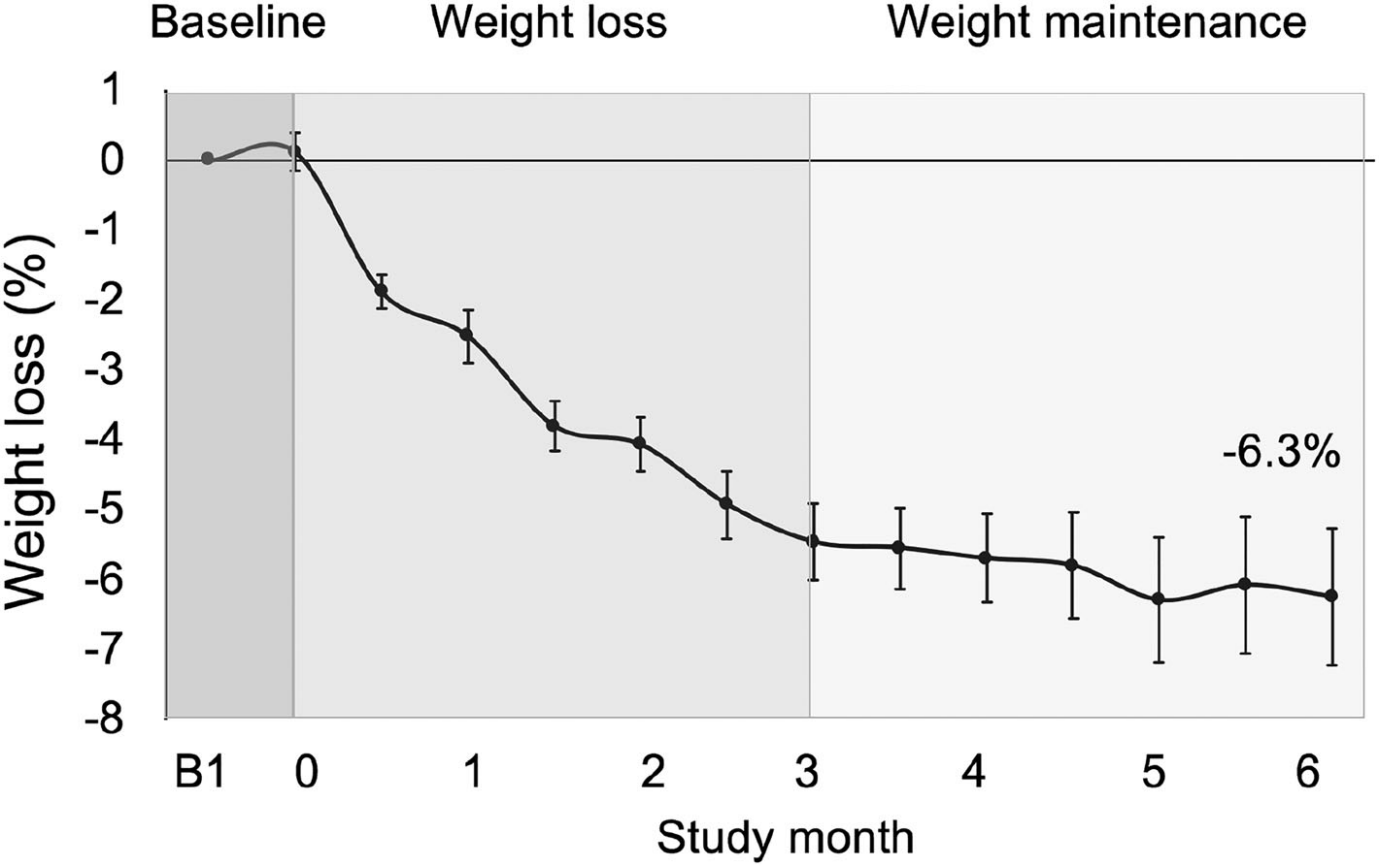
Weight loss after 6 months of alternate day fasting with a low‐carbohydrate diet. Values reported as mean ± SEM. Body weight decreased (*P* < .001) during the weight loss period (baseline to month 3) but remained stable (*P* = .57) during the weight maintenance period (months 3 to 6): Repeated‐measures ANOVA

Weight loss over the course of the trial is reported in Figure 2. Body weight remained stable (*P* = .73) from the beginning (100 ± 3 kg) to the end (100 ± 3 kg) of the baseline control period. Body weight decreased (−5.5 ± 0.5%; *P* < .001) during the weight loss period (baseline to month 3), but remained stable (*P* = .57) during the weight maintenance period (months 4 to 6). Net weight loss by month 6 was −6.3 ± 1.0%. Changes in body composition are reported in Table 3. Fat mass decreased (*P* < .01) from baseline to month 3 and from baseline to month 6. Lean mass decreased (*P* = .01) by month 3 but was not statistically different from baseline by month 6. Visceral fat mass remained unchanged over the course of the trial.

**Table 3:** Body composition and metabolic disease risk factors after 6 months of alternate day fasting with a low‐carbohydrate diet

Variable	Month 0	Month 3	Month 6	*P* value
Fat mass, kg	46 ±2	42 ±2^a^	41 ±3a	<.01
Lean mass, kg	49 ±2	48 ±1^a^	49 ±2	.01
Visceral fat mass, kg	1.4 ±0.1	1.2 ±0.1	1.2 ±0.1	.57
Total cholesterol, mg/dL	204 ±6	196 ±6	192 ±6^a^	.01
LDL cholesterol, mg/dL	123 ±5	119 ±5	113 ±5^a^	.02
HDL cholesterol, mg/dL	59 ±2	55 ±2^a^	57 ±2	.01
Triglycerides, mg/dL	118 ±11	111 ±9	104 ±9	.35
Systolic BP, mm Hg	125 ±3	121 ±3	118 ±3^a^	.03
Diastolic BP, mm Hg	85 ±3	80 ±2^a^	81 ±2	.03
Heart rate, bpm	72 ±2	72 ±2	70 ±2	.53
Fasting glucose, mg/dL	93 ±2	95 ±2	93 ±2	.84
Fasting insulin, μIU/mL	17 ±2	14 ±2^a^	13 ±2^a^	.03
Insulin resistance (HOMA‐IR)	3.9 ±0.4	3.4 ±0.4	3.2 ±0.5	.09
HbA1c, %	5.7 ±0.1	5.7 ±0.1	5.7 ±0.1	.89

*Note*. Values reported as mean ±SEM. *P* value: Repeated measures ANOVA.

Abbreviations: HbA1c, haemoglobin A1C; HDL, high‐density lipoprotein; HOMA‐IR, homeostatic model assessment of insulin resistance; LDL, low‐density lipoprotein.

a Significantly different from baseline. No significant differences between months 3 and 6 for any parameter.

### 3.3 Meal replacement adherence, dietary intake, and physical activity

On average, subjects consumed 89% of the shakes prescribed on the fast days and 85% of the shakes prescribed on the feast days. This level of adherence remained stable (*P* = .72) over the course of the 6‐month trial. Reported dietary intake is reported in Table 4. Energy intake decreased (*P* = .01) by month 3 (feast and fast day) and month 6 (feast and fast day), relative to baseline. During the weight loss period, subjects consumed 587 ± 110 kcal more than prescribed on the fast day. During the weight maintenance period, subjects consumed 676 ± 148 kcal more than prescribed on the fast day. Protein intake increased (*P* < .01) and carbohydrate intake decreased (*P* = .02) by month 3 (feast and fast days) and month 6 (feast and fast days), relative to baseline. Fat intake did not change during the trial. Cholesterol intake decreased (*P* < .01) by months 3 and 6 (fast days only) relative to baseline. Fibre intake decreased (*P* < .01) by month 3 (feast and fast days) and month 6 (feast and fast days), relative to baseline. Physical activity, measured as steps/day, did not change (*P* = .23) over the course of the study (baseline: 6931 ± 842 steps per day; month 3: 8070 ± 989 steps per day; month 6: 7648 ± 773 steps per day).

**Table 4:** Reported dietary intake during the 6‐month trial

		Feast Day			Fast Day		
Nutrient	Baseline	Month 3	Month 6	*P* value	Month 3	Month 6	*P* value
Energy, kcal	2355 ± 233	1649 ± 85^a^	1675 ± 119^a^	.01	1187 ± 111^a^	1276 ± 97^a^	.01
Protein, % kcal	16 ± 2	32 ± 1^a^	31 ± 1^a^	<.01	35 ± 2^a^	35 ± 2^a^	<.01
Carbohydrates, % kcal	50 ± 6	35 ± 2^a^	36 ± 2^a^	.02	32 ± 2^a^	34 ± 2^a^	.02
Fat, % kcal	34 ± 6	33 ± 2	33 ± 1	.11	33 ± 1	31 ± 1	.20
Cholesterol, mg	371 ± 38	305 ± 30	329 ± 42	.39	229 ± 29^a^	219 ± 25^a^	<.01
Fibre, g	20 ± 2	9 ± 1^a^	11 ± 2^a^	<.01	6 ± 1^a^	8 ± 1^a^	<.01

*Note*. Values reported as mean ± SEM. *P* value: Repeated measures ANOVA.

a Significantly different from baseline. No significant differences between months 3 and 6 for any parameter.

### 3.4 Metabolic disease risk factors

Changes in plasma lipids, blood pressure, and glucoregulatory factors are displayed in Table 3. Total cholesterol and LDL cholesterol levels remained unchanged by month 3 but were reduced (*P* < .05) by 6 ± 2% and 8 ± 3%, respectively, by month 6. HDL cholesterol levels decreased (*P* = .01) after the weight loss period (month 3) but were not statistically different from baseline by the end of the weight maintenance period (month 6). Triglyceride levels did not change over the course of the trial. Systolic blood pressure remained unchanged by month 3 but was reduced (*P* = .03) by −7 ± 3 mm Hg by month 6. Diastolic blood pressure decreased (*P* = .03) after the weight loss period (−5 ± 3 mm Hg) but was not statistically different from baseline by the end of the weight maintenance period. Heart rate did not change by month 3 or 6 of the intervention. Fasting insulin was significantly reduced (*P* = .03) by month 3 (−16 ± 6%) and month 6 (−24 ± 8%) relative to baseline. Fasting glucose, IR (measured by HOMA‐IR), and HbA1c did not change during the study.

## 4 DISCUSSION

This study examined the effects of ADF combined with a low‐carbohydrate diet on body weight and metabolic disease risk factors in individuals with obesity. The diet produced clinically meaningful weight loss over a 3‐month period and facilitated weight maintenance. In addition, improvements in several metabolic disease risk indicators, including LDL cholesterol, blood pressure, and fasting insulin were observed.

Subjects participating in the ADF low‐carbohydrate meal replacement regimen lost 5.5% of body weight after 3 months of intervention and restricted energy by approximately 35%. To our knowledge, this is the greatest degree of weight loss and energy restriction reported for ADF after this duration of time. For instance, in the studies by Trepanowski et al^7^ and Bhutani et al^10^ mean weight loss was 3% to 4% and energy restriction was 20% to 25%, after 3 months of ADF with a high‐carbohydrate diet. It is likely that the carbohydrate restricted diet in the present study played a key role in facilitating this superior degree of weight reduction and energy restriction. Accumulating evidence suggests that low‐carbohydrate diets (20%‐35% energy as carbohydrates) produce greater weight loss than traditional high‐carbohydrate diets (50%‐60% energy as carbohydrates).^13^, ^14^, ^23^, ^24^ The inclusion of meal replacements may have also contributed to this effect. Incorporating liquid meals can help participants meet daily calorie goals and macronutrient targets, which can enhance weight loss.^21^, ^22^

This study is also the first to show that ADF with a low‐carbohydrate diet can facilitate weight maintenance. By month 6, body weight was further reduced to 6.3%, although this was not statistically significant versus month 3. Only one other study^7^ has examined the ability of ADF to sustain body weight reductions. During a 6‐month weight maintenance period, Trepanowski et al^7^ observed a weight regain of approximately 1% with an ADF high‐carbohydrate diet. Taken together, the low‐carbohydrate version of ADF diet appeared to be just efficacious at helping individuals maintain weight loss as the high‐carbohydrate version of the diet.

The meal replacement regimen was generally well tolerated, although a few minor issues were reported (mild constipation and bloating). Adherence to the shake regimen was high (80%‐90% of shakes were consumed) amongst those who completed the study. However, there was a high dropout rate (40%) during the trial, particularly in the first few months of intervention. The main reason for subject attrition was dislike of the ADF regimen and/or the taste of the meal replacements. It is possible that if the subjects were provided with a wider range of meal replacement products, compliance with the diet may have been higher. It is also likely that only a selected group of individuals with obesity may find this diet tolerable and suitable for their lifestyles.

The goal in designing the ADF low‐carbohydrate intervention was to limit carbohydrate intake to 30 g/d on fast days. Results from a recent meta‐analysis^16^ indicate that consuming less than 50 g/d of carbohydrates decreases hunger and desire to eat. As such, we assumed that limiting carbohydrate consumption to 30 g would increase adherence to the fast day calorie goals as the subjects would not feel as hungry. Unfortunately, this was not the case in the present study, as subjects consumed an additional 600 calories on the fast day.

Several cardiovascular benefits were noted in the present study. After 6 months of ADF with a carbohydrate‐restricted diet, blood pressure decreased by 7 mm Hg. Blood pressure reductions (−5‐10 mm Hg) are commonly reported in ADF studies after 2 to 6 months of intervention.^10^, ^11^, ^12^, ^25^ Thus, the present findings are in line with what has been demonstrated previously. Decreases in LDL cholesterol levels (8% from baseline) after 6 months of treatment were also observed. LDL cholesterol has been shown to decrease by 10% to 20% in some,^9^, ^11^, ^25^, ^26^ but not all,^7^, ^10^, ^12^ studies of ADF. The impact of carbohydrate restriction on LDL cholesterol levels is heavily debated. While some systematic reviews and meta‐analyses show that low‐carbohydrate diets increase levels of this lipoprotein,^13^, ^27^ other show reductions^28^ or no change.^18^ Because of the design of the present study, it is not possible to ascertain whether the improvements in circulating LDL cholesterol were driven by fasting versus carbohydrate restriction. Triglyceride concentrations, on the other hand, did not change significantly over the course of the trial. This is surprising as reductions in LDL cholesterol levels are generally accompanied by decreases in triglycerides, owing to their shared metabolic pathway.^29^

The impact of this diet on glucoregulatory factors was also examined. Fasting insulin decreased by 24% after 6 months of intervention. Intermittent fasting has been shown to have potent effects on fasting insulin.^30^ More specifically, results from recent trials suggest that ADF lowers insulin levels by 20% to 60% during short‐term interventions.^7^, ^8^, ^10^, ^11^ In contrast, low‐carb diets generally have little effect on circulating insulin levels.^28^ Thus, it is possible that the reductions in insulin observed here were primarily mediated by ADF rather than carbohydrate restriction. Other markers of glucoregulation, such as fasting glucose, IR, and HbA1c were also measured. No statistically significant changes were noted relative to baseline for any of these parameters. Fasting glucose generally does not change with intermittent fasting.^30^ IR, on the other hand, is typically reduced by 20% to 40% with ADF.^30^ It remains unclear why IR was not reduced in the present trial since degree of weight loss was similar to previous trials, which showed a beneficial effects.^30^ HbA1c also remained unchanged after 6 months of treatment. However, it should be noted that HbA1c at baseline was within the normal range (ie, less than 5.7%).^31^

This study has several limitations. First, the trial did not include a no‐intervention control group. A randomized controlled trial is needed to truly assess the efficacy of this diet on body weight and metabolic disease risk. Second, the dropout rate was high (40%). In comparison, dropout rates for other ADF studies of the same duration range from 20% to 30%.^7^, ^9^ The main reason for subject attrition was dislike of the ADF regimen or meal replacement protocol. This finding suggests that ADF with a low‐carbohydrate diet may be less tolerable than traditional ADF. Third, since the subjects were consuming approximately 1200 kcal on fast days and approximately 1600 kcal on feast days, this diet more closely resembles daily calorie restriction than ADF. This should be taken into consideration when interpreting these findings. Fourth, 7‐day food records were used to assess dietary intake over the course of the trial. It is well established that individuals with obesity underreport food intake by 20% to 40%^32^, ^33^; thus, the estimates of energy intake are most likely inaccurate. This is particularly apparent during the weight maintenance phase of the trial where subjects reported an approximately 40% energy deficit versus baseline, but weight loss was limited. It is likely that by the end of the intervention, subjects were tired of documenting their food and perhaps were not as careful at recording all food items. Fifth, we only provided one type of meal replacement during the 6‐month study (ie, shakes). Providing other types of meal replacement products (eg, low‐carbohydrate bars, and soups) may have improved compliance as this would have increased diet variety.

## 5 CONCLUSIONS

In summary, these findings suggest that ADF combined with a low‐carbohydrate diet is effective for weight loss, weight maintenance, and improving certain metabolic disease risk factors such as LDL cholesterol, blood pressure, and fasting insulin. While these preliminary findings are promising, they still require confirmation by a larger‐scale randomized control trial.

## CONFLICT OF INTEREST

K.A.V. has a consulting relationship with the sponsor of the research, Nestle Health Sciences.

## FUNDING

Nestle Health Sciences

## ETHICAL APPROVAL AND CONSENT TO PARTICIPATE

The experimental protocol was approved by the Office for the Protection of Research Subjects at the University of Illinois, Chicago. All volunteers gave written informed consent to participate in the trial. All methods were performed in accordance with the relevant guidelines and regulations stipulated by the Office for the Protection of Research Subjects at the University of Illinois, Chicago.

## AUTHORS' CONTRIBUTIONS

F.K. conducted the clinical trial, analysed the data and wrote the manuscript. K.G., S.C., E.W., M.E., M.S., and V.P. assisted with the conduction of the clinical trial and data analysis. K.A.V. designed the experiment, analysed the data, and helped prepare the manuscript.

## AUTHORS' INFORMATION

Not applicable.
